# Anti-Proliferative and Apoptosis-Inducing Effect of Theabrownin against Non-small Cell Lung Adenocarcinoma A549 Cells

**DOI:** 10.3389/fphar.2016.00465

**Published:** 2016-12-02

**Authors:** Feifei Wu, Li Zhou, Wangdong Jin, Weiji Yang, Ying Wang, Bo Yan, Wenlin Du, Qiang Zhang, Lei Zhang, Yonghua Guo, Jin Zhang, Letian Shan, Thomas Efferth

**Affiliations:** ^1^Institute for Cell-Based Drug Development of Zhejiang Province, S-Evans Biosciences, Ltd.Hangzhou, China; ^2^Institute of Orthopaedics and Traumatology, Zhejiang Chinese Medical UniversityHangzhou, China; ^3^School of Medicine, Zhejiang UniversityHangzhou, China; ^4^Theabio Co., LtdHangzhou, China; ^5^Department of Pharmaceutical Biology, Institute of Pharmacy and Biochemistry, Johannes Gutenberg UniversityMainz, Germany

**Keywords:** lung cancer, A549 cells, theabrownin, apoptosis, P53

## Abstract

With the highest cancer incidence rate, lung cancer, especially non-small cell lung cancer (NSCLC), is the leading cause of cancer death in the world. Tea (leaves of *Camellia sinensis*) has been widely used as a traditional beverage beneficial to human health, including anti-NSCLC activity. Theabrownin (TB) is one major kind of tea pigment responsible for the beneficial effects of tea liquor. However, its effect on NSCLC is unknown. The aim of the present study was to evaluate anti-proliferative and apoptosis-inducing effect of TB on NSCLC (A549) cells, using MTT assay, morphological observation (DAPI staining), *in situ* terminal deoxynucleotidyl transferase dUTP nick end labeling (TUNEL) assay, and annexin-V/PI flow cytometry. Subsequently, the expression of several genes associated with cell proliferation and apoptosis were detected by real time PCR assay to explore its potential underlying mechanism. TB was revealed to inhibit cell proliferation of A549 cells in a concentration-dependent and time-dependent manner. Morphological observation, TUNEL assay and flow cytometric analysis evidenced an apoptosis-inducing effect of TB on A549 cells in a concentration-dependent manner. The real time PCR assay demonstrated that TB down-regulated the expression of *TOPO I, TOPO II*, and *BCL-2*, and up-regulated the expression of *E2F1, P53, GADD45, BAX, BIM*, and *CASP 3,7,8,9*, which suggests an activation of P53-mediated apoptotic (caspase-dependent) pathway in response to TB treatment. The western blot analysis showed a similar trend for the corresponding protein expression (P53, Bax, Bcl-2, caspase 3,9, and PARP) and further revealed DNA damage as a trigger of the apoptosis (phosphorylation of histone H2A.X). Accordingly, TB can be speculated as a DNA damage inducer and topoisomerase (Topo I and Topo II) inhibitor that can up-regulate *P53* expression and subsequently modulate the expression of the downstream genes to induce cell proliferation inhibition and apoptosis of A549 cells. Our results indicate that TB exhibits its anti-NSCLC activity via a P53-dependent mechanism, which may be a promising candidate of natural product for anti-cancer drug development in the treatment of NSCLC.

## Introduction

Owing to the deteriorating environment and people’s unfavorable living habits, lung cancer becomes the highest incidence of cancer cases and leading cause of cancer-related mortality world-wide, affecting nearly 1.38 million deaths annually (Ferlay et al., 2010). It occurs frequently in both males and females, with ∼520 000 new cases generated and 450 000 fatalities for each year in China (Tan et al., 2011). As the main type of lung cancer, non-small cell lung cancer (NSCLC) comprising 80∼85% of lung cancer cases has an extremely poor 5-year survival rate of <15%, with only a 5∼10% survival rate for advanced NSCLC (Pore et al., 2013). Besides surgery, the widely used radiotherapy and chemotherapy have been self-limited due to their unsatisfactory efficacy as well as serious side effects (Niyazi et al., 2011; Iwamoto, 2013). Therefore, it is urgently required to seek for more effective and safe agents to improve the outcome of NSCLC patients. There has been a growing interest in discovery and development of natural resources derived anti-cancer agent for their diverse biological activities and low toxicity, which is promising to provide a favorable option for NSCLC patients (Bae et al., 2015; Guo et al., 2016; Shi et al., 2016; Xing et al., 2016).

Tea is the most traditional and commonly consumed beverage for health promotion purposes in the world, produced from the fresh leaves of *Camellia sinensis* (L.) O. Kuntze (Theaceae). Apart from the health promotion effects, tea can also exert medicinal effects against many diseases, such as cancer, hyperlipidaemia, atherosclerosis, stroke, coronary heart disease, and intestinal inflammation (Khan and Mukhtar, 2007, 2008; Chen et al., 2008; Butt and Sultan, 2009). Theabrownin (TB), theaflavin (TF), and thearubigin (TR) are the three main tea pigments together determine the color, taste, as well as the beneficial effects of tea liquor (Roberts et al., 1957). TB is a kind of reddish-brown material, which can be dissolved in water other than in ethyl acetate, *n*-butyl alcohol, or other organic solvents. It comprises a family of complex macromolecules, probably formed from TF and TR by transformation of polyphenols and other bioactive components (catechins, TF, theaflavin-3′-gallate, and theaflavin-3′-3′digallate; Yang et al., 2000). TB has a significant cholesterol-lowering effect in relieving fatigue and reducing blood lipid levels (Gong et al., 2010). Since the medicinal effects of tea consumption on many human cancer types, including NSCLC, has been considered (Yang et al., 2000; Suqanuma et al., 2011), it can be expected that tea’s major active component, TB, has such an anti-cancer potential. But to date, such activity of TB is unbeknown.

For exploring the anti-cancer effect of TB against NSCLC, this study employed human lung adenocarcinoma cell lines (A549, H1299, H1650, H358, HCC827), which constitutes 80% of lung cancer cases, to evaluate TB-induced anti-proliferative and apoptosis outcome in NSCLC cells from cellular and molecular aspects. For the first time, our result would not only determine the anti-tumor activity of a tea-derived bioactive component, but also reveal its anti-NSCLC mechanism. It would contribute to the development of a new anti-cancer agent of natural product for lung cancer treatment.

## Materials and Methods

### Chemicals and Reagents

Theabrownin (>90% of purity) was purchased from Theabio Co., Ltd (Hangzhou, China; Batch number: 20151105001). Roswell Park Memorial Institute (RPMI) 1640 medium, fetal bovine serum (FBS), and 0.25% trypsin were purchased from Gibco BRL (Grand Island, NY, USA). 3-(4,5-dimethylthiazol-2-yl)-2,5-diphenyltetrazolium bromide (MTT) and dimethyl sulfoxide (DMSO) were purchased from Sigma (St. Louis, MO, USA). Annexin-V:FITC apoptosis detection kit and cell cycle kit were purchased from BD Biosciences (San Jose, CA, USA). All antibodies were purchased from Cell Signaling Technology (CST, Danvers, MA, USA). Trizol reagent and real time polymerase chain reaction (real time PCR) kit were purchased from TaKaRa (Dalian, China).

### Cell Line and Culture

Human NSCLC cell lines (A549, H1299, H1650, H358, HCC827) were obtained from Shanghai Cell Bank of Chinese Academy of Sciences (Shanghai, China) and cultured in RPMI-1640 medium containing 10% FBS at 37°C in a humidified 5% CO_2_ incubator. The medium was changed daily and the cells were treated with TB in their logarithmic growth phase.

### Cell Viability Assay

Cell viability of TB-treated NSCLC cells was determined by MTT assay method. Cells were seeded on 96-well plates with density of 5 × 10^3^ cells/well in 200 μl medium for 24 h and then treated with TB at different concentrations (0, 5, 25, 50, 100, 150, 200, 300 μg/ml) for 24, 48, and 72 h. Each 20 μl MTT solution (5.0 mg/ml) was added to each well and incubated at 37 for 4 h. Then 150 μl DMSO was added in each well to dissolve the MTT formazan crystals and the optical density value (OD value) was measured at 490 nm with a microplate reader (Biorad, Hercules, CA, USA). Inhibitory rate (%) = [1 - (TB-treated OD/untreated OD)] × 100%. The 50% inhibitory concentrations (IC_50_) for 24, 48, and 72 h were calculated by regression analysis, respectively. Then the most sensitive cell line (A549) to TB treatment was screened out for the following tests, and the low, medium, high concentrations of TB were determined.

### Cell Morphology and DAPI Staining

Theabrownin-treated A549 cells in 96-well plates were washed with phosphate-buffered saline (PBS) thrice and fixed with 4% paraformaldehyde in PBS buffer for 30 min at room temperature. Cells were stained with DAPI for 10 min in dark and then washed thrice. The unstained and stained cells were observed under a fluorescence microscope (Carl Zeiss, Göttingen, Germany). Five coverslips were used as replicates in each group and the apoptotic nuclei of each cells was visualized.

### TUNEL Assay

*In situ* observation of apoptotic cells were conducted by *in situ* terminal deoxynucleotidyl transferase dUTP nick end labeling (TUNEL) assay using *in situ* cell death detection kit, POD (Roche, Mannheim, Germany). Briefly, A549 cells were fixed with fixation solution for 1 h at 25°C and incubated in permeabilisation solution for 2 min on chamber slides. TUNEL reaction mixture was added on slides and incubated with lid for 60 min at 37°C in the dark. Afterward, the samples were analyzed in a drop of PBS under a fluorescence microscope (Carl Zeiss, Göttingen, Germany) using excitation wavelength of 450–500 nm and detection wavelength of 515–565 nm.

### Flow Cytometry

Cell apoptosis was determined by flow cytometry using an Annexin-V/PI method, according to the manufacturer’s protocol. Briefly, A549 cells were seeded on 6-well plates with density of 3 × 10^5^ cells/well for 24 h and then were treated with TB at low, medium, high concentrations for another 48 h. Afterward, the cells were harvested and washed twice with cold PBS, and labeled with Annexin V-fluorescein isothiocyanate solution and propidium iodide (PI) in binding buffer. Fluorescence intensity of the cells was detected by flow cytometry (Beckman Coulter, USA). The analysis was replicated thrice and the apoptosis rate (%) for each TB treatment was obtained.

### Real Time PCR (qPCR) Analysis

After TB treatment, gene expressions in A549 cells were detected by qPCR assay on an ABI QuantStudio^TM^ 7 Flex Real-Time PCR System (Applied Biosystems, Carlsbad, CA, USA). The total RNA of the cells in each group was extracted using Trizol reagent and synthesized to cDNA via reverse transcription. qPCR reaction system had a 20.0 μl volume: 10.0 μl SYBR^®^ Premix Ex Taq II (Tli RnaseH Plus), 0.8 μl PCR Forward Primer, 0.8 μl PCR Reverse Primer, 2.0 μl template cDNA, 0.4 μl ROX Reference Dye, and 6.0 μl ddH_2_O. The qPCR reaction condition was set to 95°C for 30 s initial denaturation, 40 cycles of 95°C for 5 s denaturation, 60°C for 34 s annealing, and 72°C for 40 s extension. At the end of each reaction, a melting curve analysis was performed. β-actin was used as the reference gene and 2^-ΔΔCT^ method was applied to analyze the relative expression of each gene (**Table 1**).

**Table 1:** Primer sequences used for qPCR analysis.

Gene	Forward primer	Reverse primer
β-actin	5′-CATGTACGTTGCTATCCAGGC-3′	5′-CTCCTTAATGTCACGCACGAT-3′
*P53*	5′-TCAACAAGATGTTTTGCCAACTG-3′	5′ -ATGTGCTGTGACTGCTTGTAGATG-3′
*BAX*	5′-CCTTTTCTACTTTGCCAGCAAAC-3′	5′ -GAGGCCGTCCCAACCAC-3′
*BCL-2*	5′-ATGTGTGTGGAGAGCGTCAACC-3′	5′ -TGAGCAGAGTCTTCAGAGACAGCC-3′
*BIM*	5′-ACCAAACCAAAGCCGTCATCA-3′	5′ -GGAGCCAGTAAACGTATTGGAAG-3′
*CASP 3*	5′-AGAACTGGACTGTGGCATTGAG-3′	5′ -GCTTGTCGGCATACTGTTTCAG-3′
*CASP 7*	5′-AGTGACAGGTATGGGCGTTCG-3′	5′ -GCATCTATCCCCCCTAAAGTGG-3′
*CASP 8*	5′-CTCCCCAAACTTGCTTTATG-3	5′-AAGACCCCAGAGCATTGTTA-3′
*CASP 9*	5′-CTGTCTACGGCACAGATGGAT-3′	5′-GGGACTCGTCTTCAGGGGAA-3′
*E2F1*	5′-CCCAACTCCCTCTACCCTTGA-3′	5′-TCTGTCTCCCTCCCTCACTTTC-3′
*GADD45*	5′-GAGAGCAGAAGACCGAAAGGA-3′	5′ -CACAACACCACGTTATCGGG-3′
*TOPO I*	5′-TCCGGAACCAGTATCGAGAAGA-3′	5′ -CCTCCTTTTCATTGCCTGCTC-3′
*TOPO II*	5′-CTAGTTAATGCTGCGGACAACA-3′	5′ -CATTTCGACCACCTGTCACTT-3′

### Western Blot Analysis

Cell proteins were extracted from the cell pellets of A549 cells using a lysis buffer (50 mM Tris-HCl pH 7.4, 150 mM NaCl, 1 mM EDTA, 1% Triton, 0.1% SDS, 5 μg/ml leupeptin, and 1 mM PMSF) for 30 min on ice repeated freezing and thawing for three times. The proteins were separated by a denaturing sodium dodecyl sulfate polyacrylamide gel electrophoresis (SDS-PAGE; 8∼12%) and then transferred onto a polyvinylidene fluoride (PVDF) membrane (Millipore, Bedford, MA, USA). The membrane was blocked with 5% non-fat milk for 2 h, followed by overnight incubation at 4°C with the primary antibodies: P53, LC3, PARP (cleavage), H2A.X (p-Ser139), Bax, Bcl-2, caspase 3 (pro- and active-), caspase 9, and β-actin. Following incubation with peroxidase-conjugated goat anti-rabbit IgG at room temperature for 2 h, proteins were visualized using enhanced chemiluminescence kit (Amersham Pharmacia Biotech, Little Chalfont, UK) and detected using a chemiluminescence analyzer.

### Statistical Analysis

Data were expressed as mean ± SD and subjected to one-way ANOVA, followed by Fisher’s least significant difference (LSD) comparison. All analyses were performed using an updated version of DPS software (Tang and Feng, 2007).

## Results

### Anti-Proliferative Effect of TB

**FIGURE 1:**
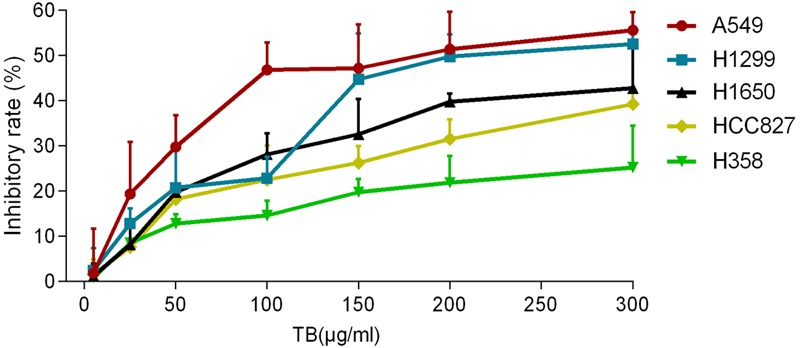
**Effect of theabrownin (TB) on cell proliferation of five non-small cell lung cancer (NSCLC) cells determined by MTT assay.** Values were presented as mean ± SD (*n* = 3).

**FIGURE 2:**
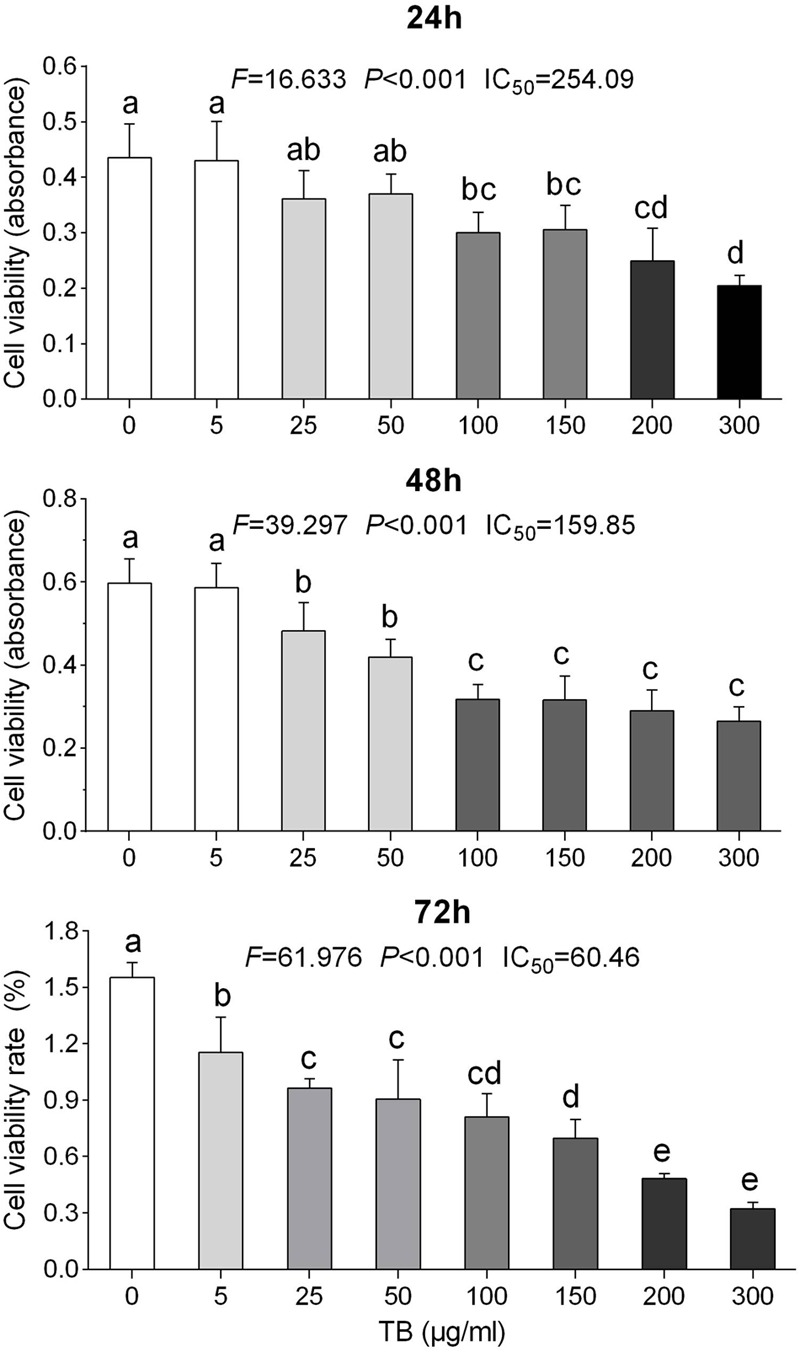
**Effect of TB on cell proliferation of A549 cells determined by MTT assay.** Values (mean ± SD, *n* = 5) with different lower case letter differed very significantly [Fisher’s least significant difference (LSD), *P* < 0.001].

To determine the effect of TB on cell growth of NSCLC, we treated five NSCLC cells lines with various concentrations (5∼300 μg/ml) of TB and conducted cell viability (MTT) assay at 48 h. As shown in **Figure 1**, the inhibitory effect on each cell line was increased with increasing TB concentrations, in which the maximum effect was observed in A549 cells. Then we performed MTT assay on A549 cells with TB treatment for 24, 48, and 72 h. The result showed that TB inhibited cell proliferation in a concentration-dependent manner at each time point (**Figure 2**). A significant inhibitory effect was found at 24 and 48 h over the concentration range from 25 to 300 μg/ml, while that at 72 h ranged from 5 to 300 μg/ml (all *P* < 0.001). The IC_50_ values declined from 254.09 to 60.46 μg/ml with increasing treatment time from 24 to 72 h, indicating a time-dependent manner of TB treatment.

### Apoptosis-Inducing Effect of TB

**FIGURE 3:**
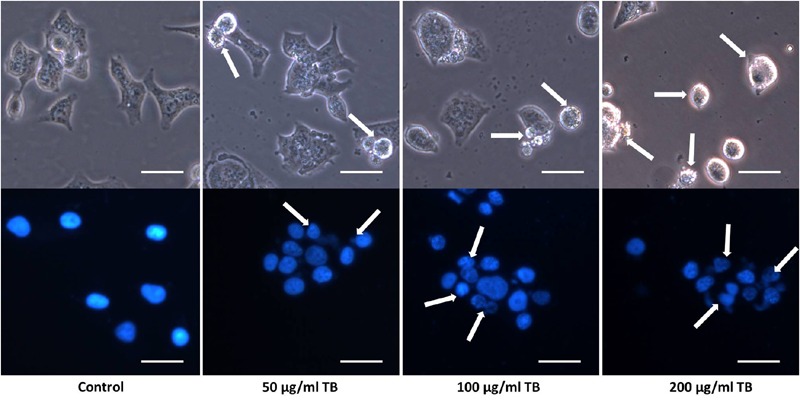
**Morphological observation on A549 cells by DAPI staining.** Scale bar: 100 μm.

Apoptotic morphological changes of A549 cells after 48 h TB treatment were observed under a fluorescence microscopy (**Figure 3**). Compared to control group, TB treated groups obviously displayed increased number of detached cells in round and shrunken shape, with increased concentration of TB in culture medium (indicated by arrows). With DAPI nucleus staining, TB treated cells showed typical apoptotic signs, including chromatin condensation, karyopyknosis, and nuclear fragmentation, which are characteristic features of apoptotic cells. In accordance with the MTT assay, a concentration-dependent manner of TB treatment was found since more apparent morphological alterations and more apoptotic cells presented with increased TB concentration.

**FIGURE 4:**
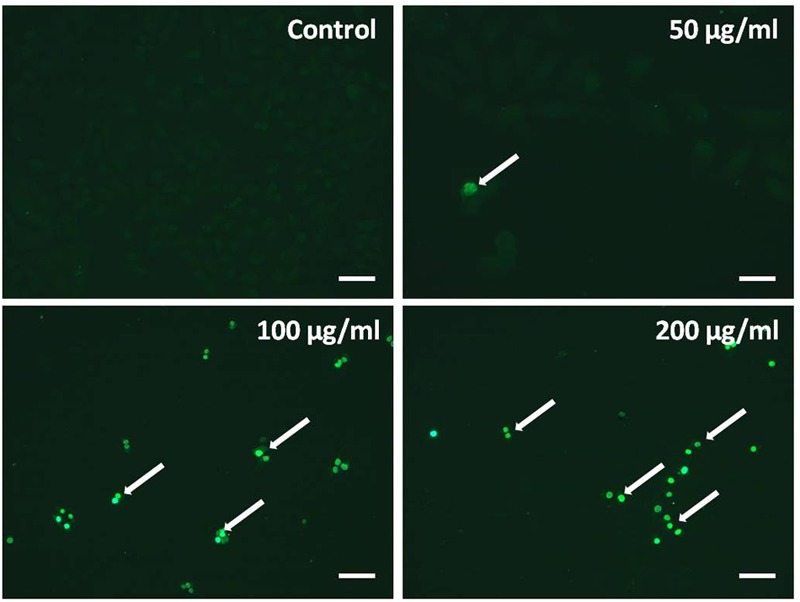
**Observation of TB induced apoptosis on A549 cells by *in situ* transferase dUTP nick end labeling (TUNEL) assay.** Scale bar: 100 μm.

Transferase dUTP nick end labeling assay was performed to evaluate TB-induced apoptosis of A549 cells. The hallmark of apoptotic cells is cell body shrinkage with nuclear chromatin condensation and DNA strand fragmentation. As shown in **Figure 4**, the control cells showed negative staining, whereas TB remarkably induced cell apoptosis with strong fluorescence staining in a concentration-dependent manner.

**FIGURE 5:**
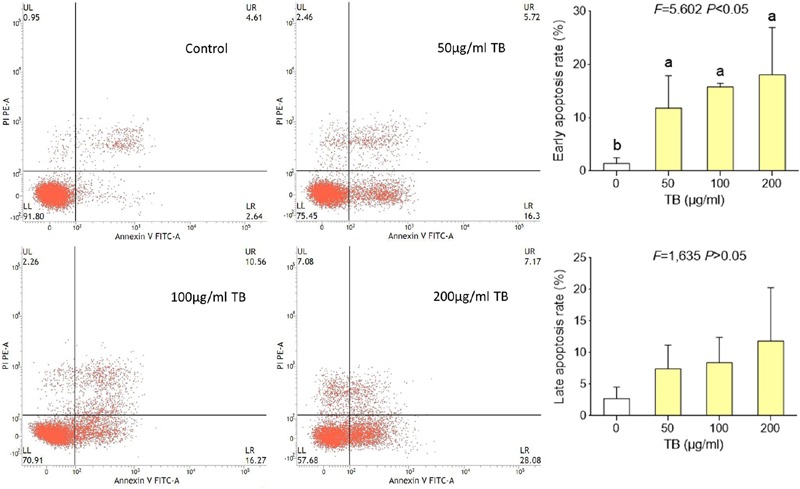
**Flow cytometry analysis of A549 cell apoptosis by double staining with Annexin V-FITC and propidium iodide (PI).** Values are presented as mean ± SD of three independent experiments. Different lower case letters indicate significant difference between groups (Fisher’s LSD, *P* < 0.001).

Flow cytometry using Annexin V-FITC/PI double staining was further performed to evidence the apoptosis-inducing effect of TB on A549 cells. As shown in **Figure 5**, significant increase of early apoptosis and progressive increase of late apoptosis were found with increasing concentration of TB treatment at 48 h. The percentages of apoptotic (early and late) cells increased from 22.5 ± 1.8% to 36.0 ± 1.8% following treatment with 50, 100, 200 μg/ml TB, respectively. It could be evidenced that TB induced apoptosis of A549 cells in a dose-dependent manner.

### Modulation on mRNA Expression by TB

**FIGURE 6:**
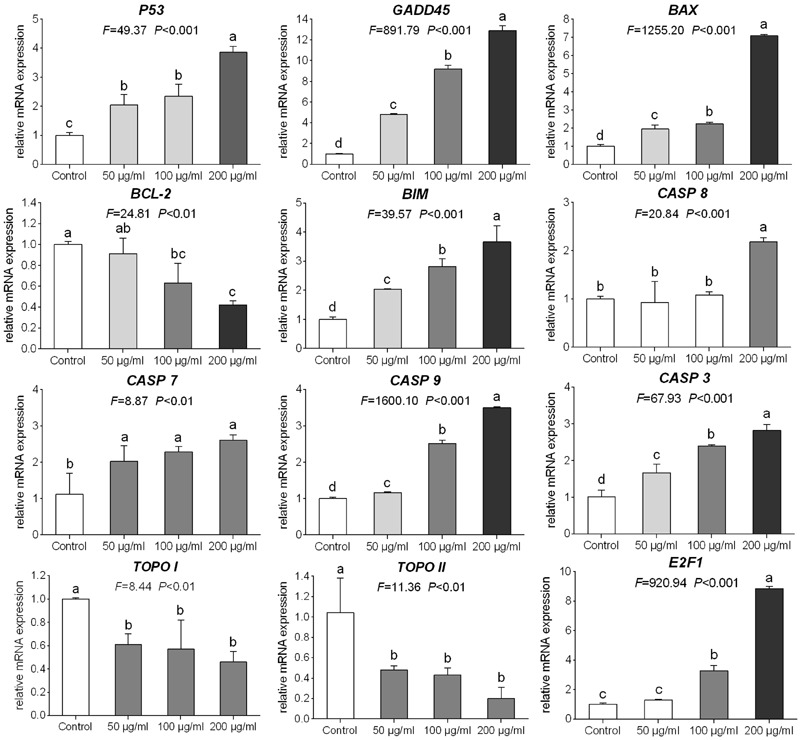
**Relative mRNA expression of target genes in A549 cells with 48 h TB treatment at 50, 100, and 200 μg/ml.** Values are presented as mean ± SD of three replicates. Different lower case letters indicate significant difference between groups (Fisher’s LSD, *P* < 0.001).

The relative expression levels of target genes modulated by TB were determined by qPCR assay. As shown in **Figure 6**, TB could significantly down-regulate the expression of *TOPO I, TOPO II*, and *BCL-2* mRNA transcripts and up-regulate the expression of *P53, GADD45, BAX, BIM, CASP 3, CASP 7, CASP 8*, and *CASP 9* mRNA transcripts in A549 cells when compared with the control group (all *P* < 0.01). In most cases, a concentration-dependent manner of TB was observed in the mRNA modulation.

### Modulation on Protein Expression by TB

**FIGURE 7:**
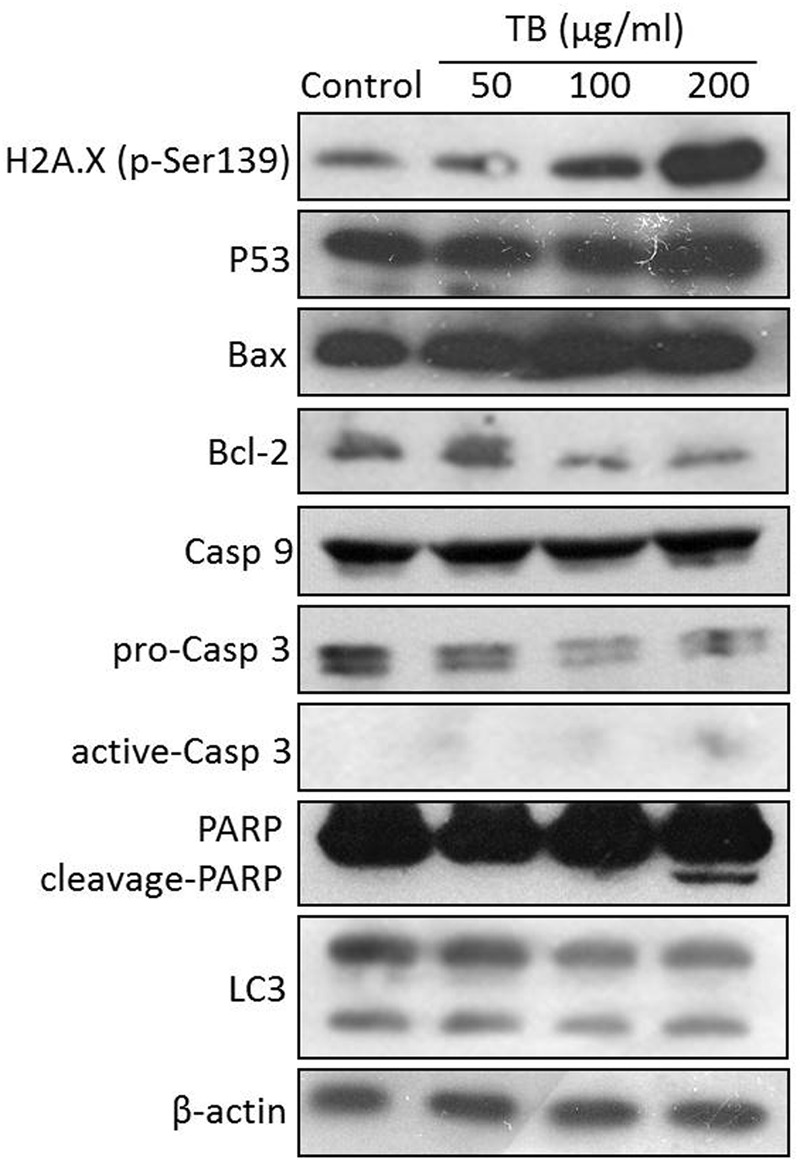
**Protein expression of target genes in A549 cells with TB treatment at 50, 100, 200 μg/ml for 48 h**.

As shown in **Figure 7**, western blot analysis showed that TB up-regulated the protein expression of H2A.X (p-Ser139), P53, Bax, caspase 9, active-caspase 3 and cleavage-PARP and down-regulated the protein expression of Bcl-2 and pro-caspase 3. The regulation effect of TB has an obvious concentration-dependent manner in most cases. Besides, TB has little effect on protein expression of LC3.

## Discussion

Dysregulation of cell proliferation and resistance to apoptosis have been verified closely associated with the progression of various types of cancer, and a number of anti-cancer treatments have been designed to suppress proliferation and induce apoptosis of tumor cells by targeting cellular processes (Hanahan and Weinberg, 2011). There has been a growing interest in the use of natural products as a new source of anti-cancer agents owing to their advantages of high bioactivities and low toxicity. Tea is such a natural product made from *Camellia sinensis* and is popularly used as beverage worldwide. Studies have indicated preventive effect of tea against various cancers, including NSCLC (Khan et al., 2009; Siddiqui et al., 2009; Yang et al., 2009). This anti-cancer effect is exerted via activation of tumor suppressor genes such as *P53*, induction of Bax/Bcl-2 mediated apoptosis, and modulation of transcription factors involved in the cancer development and progression (Rahmani et al., 2015). Although an increasing data have supported the promising anti-cancer effect of tea, many questions remain with regard to tea’s cancer-therapeutic effect and its underlying mechanism. As a representative tea component, TB was selected for evaluation of anti-cancer effect and mechanism by this study.

Our results demonstrated that TB significantly inhibited cell proliferation of NSCLC cells and had the strongest inhibitory effect against A549 cells in a concentration-dependent and time-dependent manner (**Figures 1** and **2**). Morphological observation, DAPI staining and TUNEL assay revealed typical cell apoptosis characteristics in TB-treated A549 cells (**Figures 3** and **4**), and Annexin-V/PI flow cytometric analysis further evidenced the apoptosis-inducing effect of TB at a concentration-dependent manner (**Figure 5**). In addition, gene expressions of Topo I/II, E2f1, Gadd45, P53, Bax, Bcl-2, Bim, and Caspase 3/7/8/9 in A549 cells, which are involved in proliferation and apoptosis-associated process, were found modulated by TB (**Figure 6**). Topo I and II (DNA topoisomerases) are nuclear enzymes for DNA synthesis and meiotic division that allow cells to manipulate the topology of intracellular DNA. These enzymes are highly expressed during periods of rapid cell proliferation or in cancer cells (including A549) and make transient single-strand DNA breaks (by Topo I) and double-strand DNA breaks (by Topo II) to solve topological problems arising during DNA replication, transcription, and recombination (Holden, 2001; Mirski et al., 2008). Inhibition of Topo I/II induces stabilization of transient Topo-DNA cleavage complex, leading to collisions of DNA replication forks (during replication) or progressing RNA polymerase molecules (during transcription) with these complexes, followed by formation of permanent DNA strand breaks (DSBs; Wu and Liu, 1997; Holden, 2001). The permanent DSBs can trigger DNA damage with induction of apoptosis, which has usually been adopted as an anti-cancer strategy by chemotherapeutics (Cheng et al., 2012). Thus, Topo I/II acted as DNA damage triggers targeted by TB in this study. Following the DNA damage, the transcriptional factor E2f1 can be activated as a tumor suppressor and function to inhibit cell proliferation, migration and invasion and induce apoptosis in cancer cells, including A549 (Muller et al., 2001; Duan et al., 2012). E2f1’s tumor suppressing effect is always exerted through P53-dependent mechanism, leading to up-regulation of *P53* expression (Dimova and Dyaon, 2005; Iaquinta and Lee, 2007). As a crucial transcription factor, P53 controls apoptosis through activating the transcription of hundreds of genes, including the members of Bcl-2 family and caspase family (Levine and Oren, 2009). Expectably, we found overexpression of not only transcriptional factor genes (*E2F1* and *P53*) but also downstream genes (*GADD45, BAX, BCL-2, BIM, CASP 7, CASP 8, CASP 9*, and *CASP 3*) in TB-treated A549 cells, suggesting a P53 pathway-mediated apoptosis induced by TB (**Figure 6**).

Such apoptosis of A549 cells might be triggered by DNA damage, owing to the significant up-regulation of H2A.X (p-Ser139) expression with TB treatment (**Figure 7**). Phosphorylation of histone H2A.X is one of the first cellular responses to DSBs, which acts as an indicator of the early DNA damage. Gadd45 is a P53-responsive stress protein inducible by DNA damage, which can facilitate topoisomerase relaxing and cleavage activity and affect nucleosome assembly and chromatin structure (Fornace, 1988). Overexpression of *GADD45* is capable of increasing cellular sensitivity to topoisomerase (e.g., Topo I and II) inhibitors by modifying DNA accessibility on damaged chromatin (Carrier et al., 1999), indicating its contribution to Topo-mediated DNA damage in this study (**Figure 6**). Bcl-2 family, composed of proapoptotic (e.g., Bax and Bim) and antiapoptotic (e.g., Bcl-2) members, is important for regulation of cell death, apoptosis, tumorigenesis, and cellular responses to anti-cancer therapy (Youle and Strasser, 2008). Proapoptotic member is a homolog of antiapoptotic member, each of which can be counteracted by the other in form of heterodimers (Burger et al., 1998). The ratio of proapoptotic and antiapoptotic members determines cell fate, survival, or death, when exposed to apoptotic stimuli (Oltvai et al., 1993). As representative proapoptotic members, Bax/Bim provokes apoptosis and cell death via activation of intrinsic caspase-dependent pathways in mitochondria, while antiapoptotic Bcl-2 inhibits apoptosis via prevention of caspase activation by suppressing cytochrome *c* release from mitochondria (Rosse et al., 1998). Since P53 can regulate those Bcl-2 members in response to a variety of apoptosis triggers, the up-regulation of Bax/Bim with down-regulation of Bcl-2 found by this study confirmed the P53-dependent mechanism of TB. Caspases (cysteine–aspartic acid proteases) are also key mediators of apoptosis, and most cell apoptosis-inducing factors eventually cause cell apoptosis through the caspase-mediated signal transduction pathway (Xie and Yang, 2014). Caspases 8 and 9 are initiating caspase proteins in the caspase cascade which can activate crucial death proteases, whilst caspase 7 is an executioner in the caspase cascade directly leading to apoptosis (Cohen, 1997). Caspases 8 and 9 both activate caspase 3 and induce subsequent cleavage of PARP to execute the apoptotic process, leading to phosphatidylserine translocation and DNA fragmentation (Yakovlev et al., 2000). Similar to proapoptotic Bcl-2 family members, caspase 9, and caspase 7 play key roles in the mitochondria-mediated intrinsic apoptosis pathway (Xiong et al., 2014). Our results showed gene up-regulation of *CASP 9, CASP 7*, and *CASP 3* as well as protein up-regulation of active-caspase 3 and cleavage-PARP with TB treatment (**Figures 6** and **7**), indicating intrinsic caspases pathway as a main pathway involved in TB-induced apoptosis of A549 cells. A significant up-regulation of *CASP 8* was seen with TB treatment at 200 μg/ml, indicating that extrinsic pathway might also be involved in TB-induced apoptosis. Our data on protein expression of LC3 further revealed that the apoptosis of A549 cells was not associated with autophagy (**Figure 7**).

**FIGURE 8:**
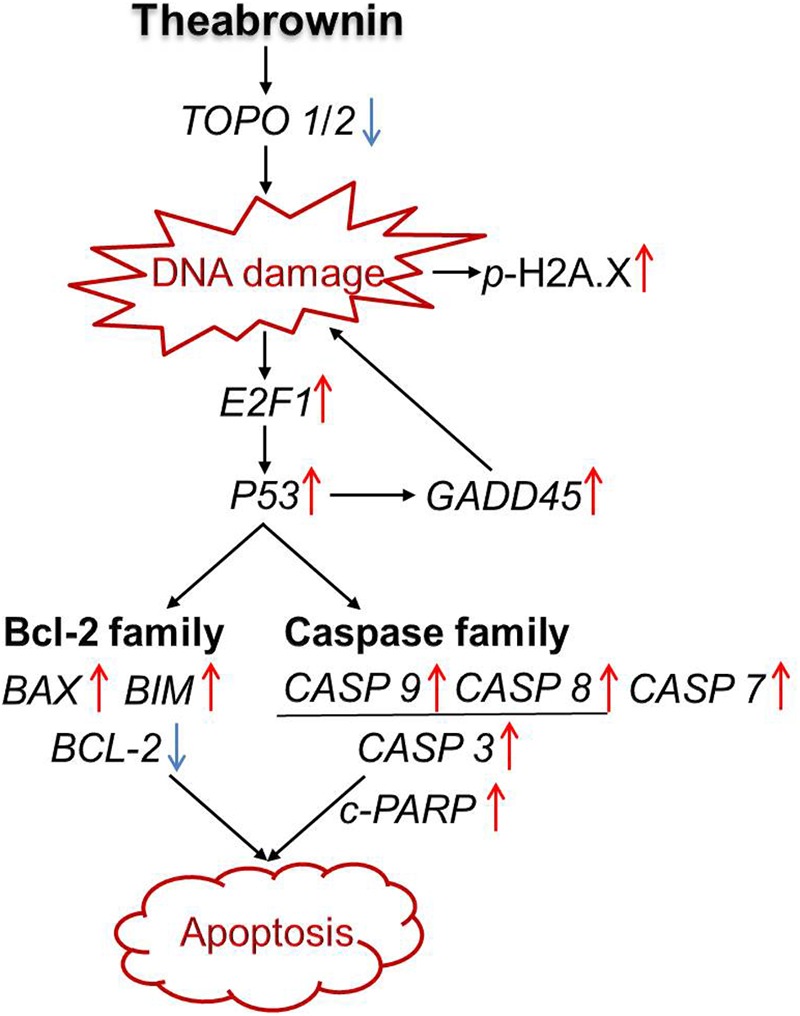
**Apoptosis-inducing mechanism of TB on A549 cells**.

Taken together, our results suggest that TB might act as a DNA damage inducer as well as topoisomerase (Topo I/II) inhibitor that has inhibited cell proliferation and induced apoptosis of A549 cells through a P53-mediated caspase-dependent mechanism (**Figure 8**). Such finding is in accordance with previous studies regarding tea’s anti-NSCLC effect and the corresponding mechanism (Yang et al., 2009; Rahmani et al., 2015), indicating TB as a representative tea component responsible for tea’s anti-cancer efficacy. Further studies are warranted to confirm TB’s *in vivo* anti-cancer efficacy on NSCLC and the corresponding molecular mechanisms. Besides, since TB is a complex polymeric polyphenol derivants, it is very possible to be degraded by the gastrointestinal tract. Therefore, further studies should also find some appropriate formulations for the development of effective products based on TB.

## Conclusion

The present study demonstrated that TB can inhibit NSCLC cell proliferation and induce apoptosis in a concentration-dependent manner. The apoptosis was triggered by DNA damage and mediated by P53-mediated caspase-dependent pathway. In this pathway, TB acted as a DNA damage inducer and topoisomerase inhibitor to up-regulate *P53* expression and subsequently modulate the expression of the downstream genes and proteins, leading to cell apoptosis that can finally induce NSCLC suppression. This study would facilitate the understanding of anti-cancer pharmacological profile of TB and contribute to the development of tea-derived anti-cancer agent for lung cancer treatment.

## Author Contributions

FW, LZ, and BY performed the main experiments of this study; WY, YW, WD, QZ, YG, and JZ contributed to the materials acquisition and data analysis for this study; LS and LZ designed this work and drafted the manuscript; TE improved the design and draft of this work; WJ contributed to the revision of this manuscript. All listed authors approved the version for publication, and agreed to be accountable for all aspects of this work.

## Conflict of Interest Statement

The authors declare that the research was conducted in the absence of any commercial or financial relationships that could be construed as a potential conflict of interest.
